# Functional siRNA Screen Links Ras/MAPK and Wnt Pathway to EV Secretion in HCT-116 Colorectal Cancer Cells

**DOI:** 10.3390/diseases14030089

**Published:** 2026-03-02

**Authors:** Sophie Marie Pätzold, Julia Christina Gross

**Affiliations:** 1 Hematology and Oncology, University Medical Center Goettingen, 37075 Goettingen, Germany; sophiem.paetzold@gmail.com; 2 Department Medicine, Institute of Molecular Medicine, HMU Health and Medical University Potsdam, 14471 Potsdam, Germany

**Keywords:** exosomes, microvesicles, oncogene, tumor suppressor gene, HCT-116-cells, EV secretion, siRNA-mediated knockdown, Ras/Raf/MAPK pathway, Wnt pathway

## Abstract

**Background**: Extracellular vesicles (EVs) play an important role in tumor progression and intercellular communication, yet the contribution of specific cancer-related genes to EV secretion remains incompletely defined. **Methods**: To address this, we performed an siRNA-based loss-of-function screen targeting 30 frequently altered (proto-)oncogenes and tumor suppressor genes in the colorectal carcinoma cell line HCT-116 to assess their impact on EV release. EVs were isolated by sequential ultracentrifugation to obtain P14 and P100 fractions pelleting at 14,000× *g* or 100,000× *g*, respectively, and were characterized by nanoparticle tracking analysis, EV marker expression, and total protein quantification. Cell viability was assessed to control for potential apoptosis-related effects. **Results**: With few exceptions, knockdown of the investigated genes led to an increase in EV secretion. Silencing of *KRAS* and *BRAF* resulted in significantly elevated P14 EV levels, increased EV marker expression, and higher total protein content, while *KRAS* knockdown was additionally associated with a shift toward larger particle sizes. Downregulation of *CTNNB1* increased P14 and decreased P100 EV secretion, whereas *CDH1* knockdown reduced P14 EV levels and slightly increased P100 EVs. No general distinction between tumor suppressor genes and (proto-)oncogenes regarding their effects on EV secretion was observed, and cell viability was not significantly altered under the experimental conditions. **Conclusions**: These findings suggest that components of the Ras/Raf/MAPK and Wnt signaling pathways may contribute to the regulation of EV secretion in colorectal cancer cells.

## 1. Introduction

Extracellular vesicles (EVs) are a highly heterogenous group of nanoparticle-sized structures surrounded by a lipid bilayer that are abundantly secreted by all types of cells into the extracellular space [1]. Initially described as a cellular mechanism for waste disposal [2], only since the 2000s has the importance of EVs became more and more evident. Due to their ability to carry specific cargo, such as proteins, lipids, and functional nucleic acids from their originating cell to more distant cells, EVs play a fundamental role in cell-to-cell communication [3]. Therefore, a variety of physiological processes have been shown to be influenced by EVs: blood coagulation [4], modulation of innate and adapted immune responses [5,6], autophagy [7], tissue repair [8], implantation [9] and pregnancy [10], homeostasis and differentiation of stem cells [11], neuronal communication within the central nervous system [12], and modulation of metabolic processes [13,14].

Based on their biogenesis and size, EVs can be divided into three major groups: exosomes or small EV (30–150 nm in diameter) and microvesicles or large EV (100–1000 nm) in viable cells, and apoptotic bodies (100–5000 nm) in cells undergoing a programmed cell death (apoptosis). Exosomes emerge in the endosomal pathway by inward budding of the endosomal membrane, creating intraluminal vesicles (ILV) and ultimately multivesicular bodies (MVB, late endosomes), which can fuse with the plasma membrane to release the intraluminal vesicles into the extracellular space as exosomes. In contrast, microvesicles are created by budding and shedding directly from the plasma membrane [3].

Since 2014, the International Society for Extracellular Vesicles (ISEV) has been publishing guidelines based on expert consensus, its Minimal Information for Studies of Extracellular Vesicles (MISEV), which represent the current state of knowledge in the research field and are intended to serve researchers as guidelines for nomenclature and methodology for the purification and analysis of EVs and for error processing [15,16,17,18]. According to the current MISEV, particles pelleting at 14,000× *g* in ultracentrifugation will be called “P14 EVs”, whereas particles pelleting at 100,000× *g* in ultracentrifugation will be referred to as “P100 EVs”.

Based on their functions in physiological processes, the interest in the role of these special particles in pathological processes as well as therapeutical use have grown rapidly. Especially in the field of oncology, EVs show great potential as possible biomarkers [19], as a therapeutical target, and as potential vehicles to deliver drugs [20].

Although singular proteins influencing EV secretion have been described, no general attempt to systematically look at tumor suppressor genes and (proto-)oncogenes and their influence on EV secretion has been made thus far.

The aim of this study was to find out whether the loss of function of different cancer-related genes, found mutated in the most common tumor entities, impact the secretion of extracellular vesicles (EV). For this purpose, 30 candidates were chosen by researching known interactions with EV in the literature, looking at known RNAi phenotypes from genome-wide studies and frequencies of gene mutations within most common tumor entities. For example, double-stranded DNA fragments harboring oncogenes have been identified in small EVs isolated from various human cancer cell lines, including colorectal cancer, using polymerase chain reaction techniques [21,22]. The upregulated oncogene MYC has been detected in large EVs isolated from tumor-bearing mice [21], whereas the BRAF V600 mutation has been identified in small EVs obtained from a melanoma cell line and from melanoma-bearing mice [23]. Furthermore, next-generation sequencing of circulating small EVs from patients with pancreaticobiliary malignancies revealed mutations in several oncogenes, including *kirsten rat sarcoma 2 viral oncogene homolog* (KRAS), *breast cancer susceptibility gene 2* (BRCA2), *epidermal growth factor receptor* (EGFR), *erythroblastic leukemia viral oncogene homolog 2* (ERBB2), and MYC [24].

Functionally relevant tumor suppressor genes, including *cadherin-1* (CDH1) and *phosphatase and tensin homolog* (PTEN), have also been associated with EVs. Downregulation of PTEN mediated by astroglia-derived exosomal microRNAs has been shown to promote brain metastasis of primary tumors [25]. Moreover, sEV-mediated, E-cadherin-dependent reduction in intracellular *β-catenin* (CTNNB1) has been described as a mechanism that antagonizes the Wnt signaling pathway, which, beyond its physiological role in embryogenesis, plays a crucial role in tumor cell proliferation [26].

To systematically investigate the impact of these cancer-related genes on EV secretion, we performed an siRNA-mediated screening approach targeting 30 selected (proto-)oncogenes and tumor suppressor genes in the colorectal carcinoma cell line HCT-116. All candidates chosen for this study are summarized in Table 1, which also depicts their mutational status in HCT-116 cells.

## 2. Materials and Methods

### 2.1. Cell Culture

The human colorectal carcinoma cell line HCT-116 (ACC 581, DSMZ.de) was cultivated in 75 cm^2^ flasks in *Dulbecco’s Modified Eagle Medium (DMEM)* containing 10% *Fetal Bovine Serum* and 1% penicillin and streptomycin. Incubation occurred in 5% humidified CO_2_ at 37 °C. Upon reaching 80% confluence, cells were split 1:10 by discharging the serum, washing with 5 mL 1× PBS, using 0.25% Trypsin EDTA for cell detachment, and finally counteracting the Trypsin activity with DMEM. Cell counting was performed using the electronic CASY cell counter. Therefore, cells were treated as previously described, diluted 1:400 in CASYton, and measured according to the manual.

### 2.2. Reverse siRNA Transfection

In order to specifically downregulate single endogenous genes, 0.4 × 10^6^ HCT-116 cells diluted in 1.5 mL DMEM per well were reverse transfected in a 6-well setup using specific siRNAs and incubated while maintaining 37 °C and 5% CO_2_ for 72 h. All siRNAs were (siTOOLs Biotech GmbH, Planegg, Germany) diluted in RNAse-free water under sterile conditions to a concentration of 10 µM upon use. siPOOLs consist of specifically designed pools of 30 individual siRNAs, all targeting different sequences within the same target gene. This strategy reduces off-target effects at very low concentration of each siRNA with the strong silencing provided by the cooperative activity of many siRNAs. An exemplary knockdown validation by Western blotting of CTNNB1 and BRAF was successful in HCT-116 cells (Supplementary Figure S1).

After 48 h, the medium was switched from DMEM to *exo-free medium* (DMEM medium ultracentrifuged at 100,000× *g* overnight, filtrated under sterile conditions, and stored at 4 °C). After 72 h, the 6 wells were put on ice and the supernatants from each well were taken for the isolation of EVs and consecutive analysis, whereas the remaining cell pellets were used to produce cell lysates to allow analysis concerning protein levels. Cells were lysed with 300 µL RIPA buffer/Protease Inhibitor Cocktail (ratio 1:1000) per well and mechanically loosened using a pipette tip. Next, the lysates were transferred into Eppendorf cups and centrifuged at 16,000× *g* and 4 °C for 5 min, before the supernatants were transferred into new cups and stored at −20 °C until further use.

### 2.3. Isolation of Extracellular Vesicles (EVs)

Ultracentrifugation was used for the isolation of EVs. Supernatants from treated HCT-116-cells were centrifuged at 750 *g* for 10 min to pellet floating cells. Afterwards, the supernatants were transferred into fresh cups and centrifuged at 1500× *g* for 10 min. Next, the supernatants were transferred into fresh cups again and centrifuged at 14,000× *g* for 35 min, leading to a pellet containing EV–P14 pellets, which were washed in 1× PBS and again centrifuged at 14,000× *g* for 35 min. The supernatants were discarded and the P14 pellets consecutively resuspended in 50 µL 1× PBS and stored at −20 °C for further analysis. (P14 EVs). The supernatants were transferred into fresh cups for ultracentrifugation at 100,000× *g*. In the same way as the ultracentrifugation at 14,000× *g*, the P14 supernatants were ultracentrifuged at 100,000× *g* for 1 h, resulting in pellets containing P100 EVs. After washing in 1× PBS and further ultracentrifugation at 100,000× *g* for 1 h, the P100 pellets were resuspended in 50 µL 1× PBS and stored at −20 °C for further analysis.

### 2.4. Nanoparticle Tracking Analysis

Nanoparticle tracking analysis (NTA) was used to characterize isolated EVs regarding concentration, size, and size distribution. Measurements took place at MPI Goettingen using the NanoSight NS300 (Malvern Panalytical, Malvern, Worcestershire, UK). Briefly, the samples were diluted 1:20 in sterile filtrated 1× PBS and transported on ice to the location. Camera and analysis settings were manually kept the same for each measurement, as shown in Table 2 and Table 3. For each biological replicate, NTA created 3 technical replicates. 

Data analysis was performed by the corresponding software Nanosight NS300 Version 3.2.16 (Malvern Panalytical, Malvern, UK).

### 2.5. Western Blot

For the detection of typical markers for EVs as well as for the quantification of specific proteins, 4–12% gels with 10–15 wells (Invitrogen AG, Carlsbad, CA, USA) were used for SDS-PAGE under reducing conditions. To each 25 µL P14 or P100 sample, 5 µL of 6× SDS buffer was added, whereas 1 µL of 6× SDS buffer was added to each 5 µL of cell lysate, and a standard 3 µL *PageRuler Plus Prestained Protein Ladder* (Thermo Fisher Scientific Inc., Waltham, MA, USA) was utilized. All samples were mixed well, incubated at 95 °C for 5 min, and shortly centrifuged before application to the gels. All gels ran for approximately 1 h at 80 V and another 30 min at 120 V. Subsequently, the PVDF membrane was incubated in blocking solution for 30 min at room temperature, washed in 1× TBST, and incubated in primary antibody overnight. The following day, the membrane was washed in 1× TBST three times for 10 min each. Afterwards, incubation in a fluorchrome-conjugated secondary antibody took place for 2 h. After another washing step in 1× TBST, imaging via Odyssey CLx Imaging System (Li-Cor, Lincoln, NE, USA) took place. Quantification of the Western blot results was performed by utilizing ImageStudioLite Version 5.2.5 (Li-Cor, Lincoln, NE, USA).

Marker detection was used to certify EV identity and purity. The presence of CD81, CD147, or Flotillin-1 for P14 and ALIX, CD63, CD81, or TSG101 for P100 and the absence of negative markers Calnexin and GM130 were selected as established EV-associated markers in accordance with MISEV recommendations [18].

### 2.6. Quantification of Total Protein

The total protein of samples containing isolated EVs was determined by Pierce BCA Protein Assays, Enhanced Protocol (Thermo Fisher Scientific Inc., Waltham, MA, USA). Therefore, HCT-116 cells were reverse-transfected with the respective siRNAs at equivalent doses to the standard experimental setup and incubated for 96 h at 37 °C and 5% CO_2_ in opaque 96-well microtiter plates to accommodate subsequent luminescence measurement. Measurements were performed in a 1:5 dilution in 1× PBS according to the manufacturer’s protocol using the spectrophotometer and corresponding software Nanodrop 2000c (Thermo Fisher Scientific Inc., Waltham, MA, USA).

### 2.7. Determination of Cell Viability

CellTiter-Glo One Solution Assay (Promega Corporation, Madison, WI, USA) was used to determine ATP-based cell viability. Measurements were performed using Luminometer Centro LB 960 (Berthold Technologies GmbH, Bad Wildbad, Germany) and MikroWin 2000 Lite Version 4.43 (Labsis Laborsysteme GmbH, Neunkirchen-Seelscheid, Germany).

### 2.8. Statistical Analysis

GraphPad Prism Version 10.2.3 (GraphPad Software Inc., Boston, MA, USA) was used for statistical analysis. Three independent experiments were performed. Data are presented as the mean ± standard deviation of the mean. The significance threshold was α = 0.05 (*p* < 0.05 = *, *p* < 0.01 = **, *p* < 0.001= ***, *p* < 0.0001 = ****). The confidence interval for the z-score was 90% (z = 1.64). Comparison between the two groups were performed using Student’s *t*-test. Multiple group comparisons were performed using one-way ANOVA and consecutive *Tukey’s multiple comparison test.* Nanoparticle tracking analysis generated 3 technical replicates for each biological replicate; thus, 9 data points are depicted in figures representing NTA data.

## 3. Results

**Figure 1:**
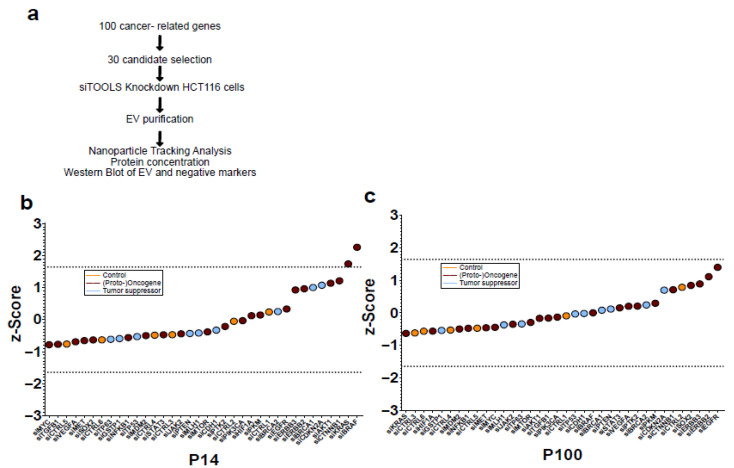
EV secretion screen against 30 cancer-related genes. (**a**) Study overview; (**b**) z-score of NTA concentrations of purified P14 and (**c**) P100 EVs after downregulation of different tumor suppressors (blue), (proto-)oncogenes (dark red), and controls (yellow) using reverse siRNA transfection in colon-cancer HCT-116 cells.

To evaluate the impact of these cancer-related genes on EV secretion, HCT-116 cells were subjected to siRNA-mediated knockdown followed by EV isolation and characterization (Figure 1a).

Since the purification of EVs is still technically prone to errors [15,28,29], the average of the three plate controls in which the target gene was located during the purification of EVs was taken as the control value instead of averaging all controls. In order to be able to classify the spread between the controls and still enable comparability of the values, the z-score was calculated. The z-score is a statistical measure that represents the deviation of an individual value from the mean of the overall distribution. A z-score of 0 corresponds to the mean of the distribution, a z-score of 1 is one standard deviation above the mean of the distribution, and a z-score of −1 is one SD below the mean of the distribution, etc. Thus, the z-score is suitable for comparing values with different scaling, as well as for identifying outliers [30]. Z-scores of NTA concentrations of purified P14 (Figure 1b) and P100 EVs (Figure 1c) after downregulation of different tumor suppressors, (proto-)oncogenes, and controls using reverse siRNA transfection in colon-cancer HCT-116 cells initially point out the target genes KRAS and BRAF to play a potential role on P14 EV secretion (Figure 1b).

### 3.1. No Generic Effects of Cancer-Related Genes on EV Secretion

**Figure 2:**
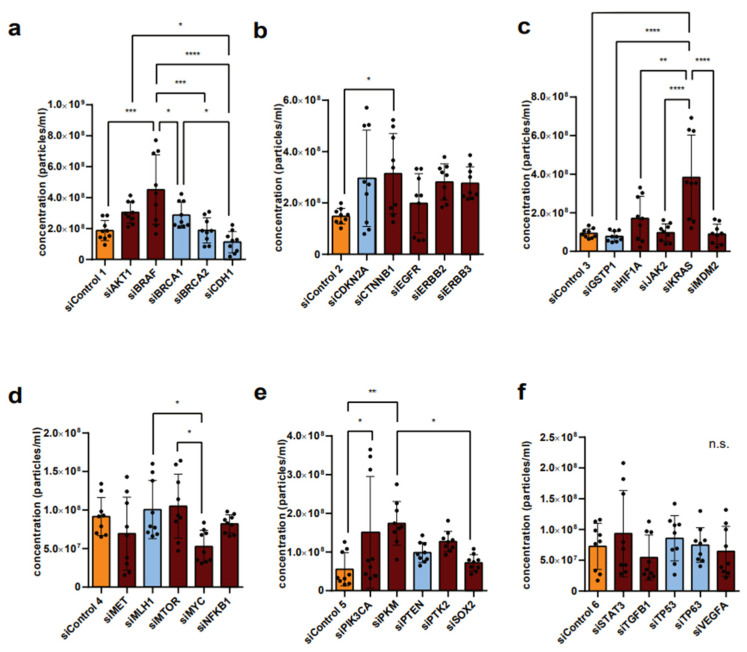
Nanoparticle analysis of P14 concentration after downregulation of various tumor suppressors and (proto-)oncogenes using siRNA in HCT-116 cells. (**a**–**f**) Tumor suppressors (blue), (proto-)oncogenes (dark red), and controls (yellow) were downregulated by siRNA in six batches in three biological replicates. Significance level: * *p* < 0.05, ** *p* < 0.01, *** *p* < 0.001, **** *p* < 0.001.

Overall, no general increase or decrease in the secretion of EVs could be observed after the knockdown of tumor suppressor genes or after the knockdown of (proto-)oncogenes. There was no visible connection between tumor suppressor genes in general and the secretion of EVs, nor between (proto-)oncogenes and the secretion of EVs in general, while the effect of individual genes was in several cases significant. Significant elevation of P14 EV secretion is shown after the downregulation of BRAF (Figure 2a), CTNNB1 (Figure 2b), KRAS (Figure 2c), and PKM (Figure 2e) in HCT-116-cells.

**Figure 3:**
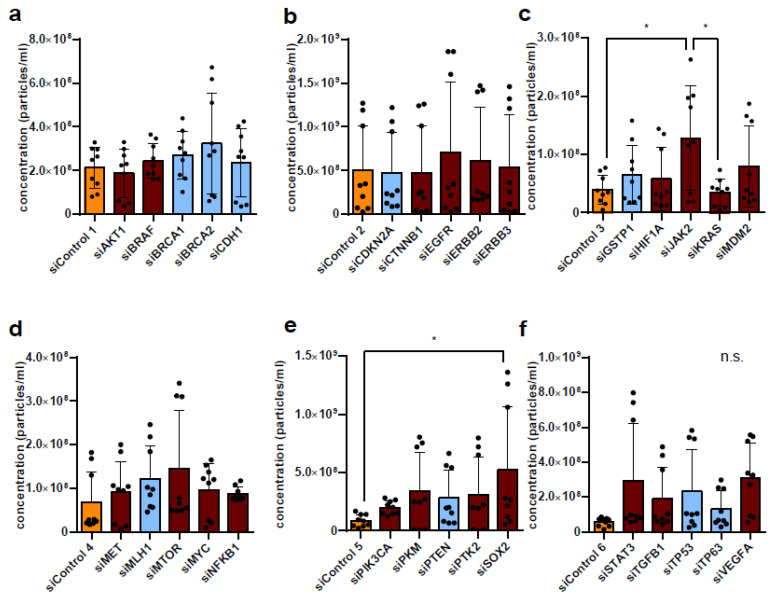
Nanoparticle determination of P100 concentration after downregulation of various tumor suppressors and (proto-)oncogenes using siRNA in HCT-116 cells. (**a**–**f**) Tumor suppressors (blue), (proto-)oncogenes (dark red), and controls (yellow) downregulated by siRNA in six batches in three biological replicates. Significance level: * *p* < 0.05.

Interestingly, the downregulation of the respective target gene predominantly led to increased secretion of EVs—regardless of whether the downregulated target gene was a tumor suppressor or (proto-)oncogene. This was true for the secretion of EVs in the P14 fraction (Figure 2a–f) as well as in the P100 fraction (Figure 3a–f). Exceptions resulting in decreased P14 EV secretion from HCT-116 cells could be seen after the downregulation of CDH1 (Figure 2a), GSTP1 (Figure 2b), MET, MYC, NFKB1 (Figure 2d), and TGFB1 (Figure 2f), but none of these decreases proved to be statistically significant. A non-significant reduction in EV secretion resulting in fewer P100 EVs was detected after the downregulation of AKT1 (Figure 3a), CDKN2A, CTNNB1 (Figure 3b), and KRAS (Figure 3c) in HCT-116 cells. Since the effects were stronger at P14 than at P100, we focused our analysis on the P14 condition.

### 3.2. KRAS Knockdown Leads to Significantly More P14 EV Secretion as Well as to Secretion of Significantly Bigger Particles

KRAS, v-Kirsten rat sarcoma 2 viral oncogene homolog, is a proto-oncogene that encodes the protein Ras. The Ras protein can bind guanosine diphosphate (GDP) and guanosine triphosphate (GTP), with the GTP-bound form representing the activated state. Ras has intrinsic GTPase activity, which can hydrolyze GTP to GDP and thus inactivate Ras. This is enhanced by GTPase-activating proteins (GAPs), whereas guanine nucleotide exchange factors (GEFs) mediate the exchange of GDP to GTP and thus activate the Ras protein [31].

As an essential component of signaling pathways, Ras proteins play an important role in the regulation of cell proliferation. The best-known signaling pathway for this is the Ras/Raf/MAPK signaling pathway. Since KRAS is a proto-oncogene, mutations in the proto-oncogene can lead to the formation of an oncogene. Up to 30% of all human malignant tumors have Ras mutations, and they are found in as many as 30–40% of colorectal carcinomas [32].

The HCT-116 cells used in this study also carry a KRAS mutation, which causes the encoded Ras protein to lose its GTPase activity, leading to permanent activation and thus constitutively promoting cell growth in the sense of the first hallmark of malignant tumors [33]. Knockdown of KRAS in HCT-116 cells leads to significantly more EVs in the P14 fraction compared to the control (RNAi using non-targeting RNA) (Figure 2c), whereas the concentration of EVs in the P100 fraction is reduced but not statistically significant (Figure 3c).

**Figure 4:**
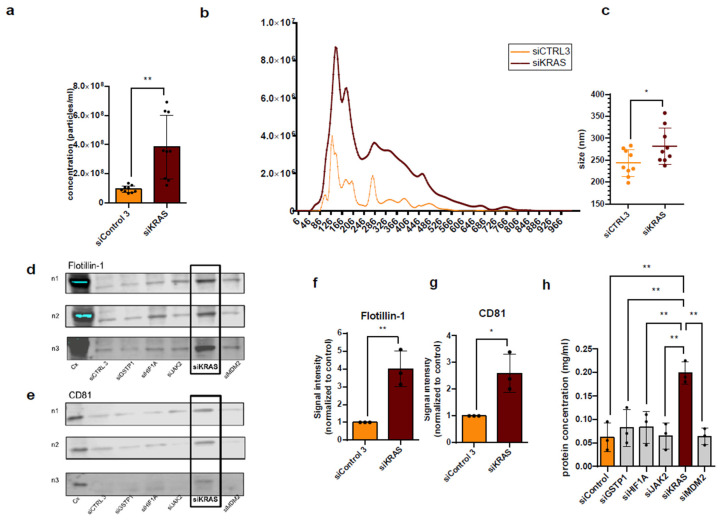
Knockdown of KRAS increases P14 EV secretion from HCT-116 cells and leads to bigger particle sizes as well as elevated levels of total protein. (**a**) Particle concentration measured by NTA shown by *t*-test. (**b**) Size distribution of P14 siKRAS and siCtrl measured by NTA. (**c**) Average sizes of P14 EVs after downregulation of KRAS compared to siCTRL, (**d**–**g**) marker distribution and quantification of Flotillin-1 (47 kDa) and CD81 (25 kDa) by Western blot, and (**h**) total protein concentration measured by BCA protein assay (enhanced protocol). Significance level: * *p* < 0.05, ** *p* < 0.01. Original images are provided in File S1.

**Figure 5:**
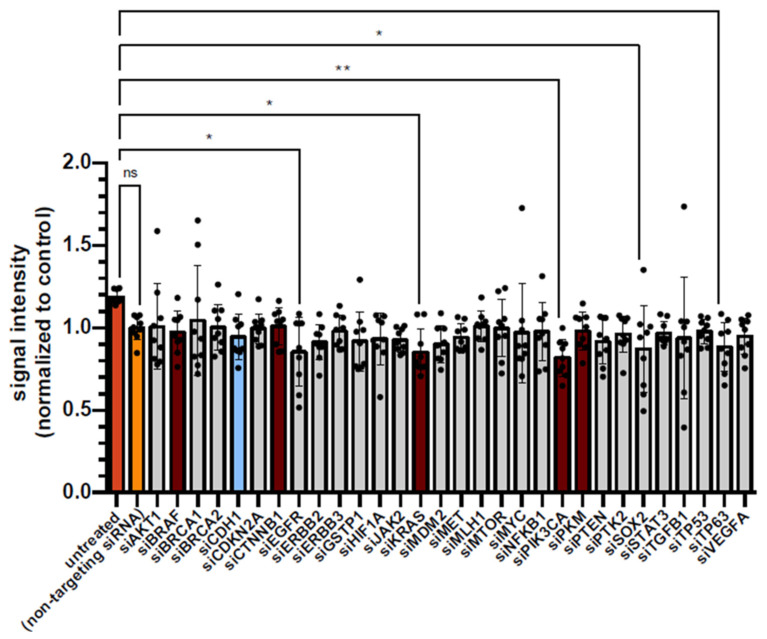
Cell viability is not changed by siTools knockdown of 30 different cancer-related genes/proteins. The determination of cell viability was performed using an ATP-based approach with the CellTiter-Glo One Solution Assay. Cell viability after downregulation of each tumor gene using siRNA in HCT-116 cells is shown normalized to HCT-116 cells treated with non-targeting siRNA. Target genes of interest are highlighted in color, whereas the remaining candidates are shown in gray. Tumor suppressors are depicted in blue, (proto-)oncogenes in dark red, controls (non-targeting siRNA) in yellow, and untreated HCT-116 cells in orange. Significance level: * *p* < 0.05, ** *p* < 0.01.

In addition to the significantly increased secretion of EVs in the P14 fraction (Figure 4a,b), after the downregulation of KRAS, there was on average a significant increase in the size of the EVs in the P14 fraction (Figure 4b,c). Flotillin-1 and CD81, two well characterized markers of EVs, were 4-fold and 3-fold increased upon KRAS KD (Figure 4d–g). Lastly, an increased total protein concentration in the P14 fraction was observed compared to the control (Figure 4h). While ATP-based viability measurements (Figure 5) did not indicate reduced cell viability, the observed increase in particle size after *KRAS* knockdown could be partially contributed to by apoptotic bodies.

### 3.3. BRAF Knockdown Leads to Increased Secretion of P14 EVs

BRAF, v-raf murine sarcoma viral oncogene homolog B1, is a proto-oncogene that encodes the protein of the same name, B-Raf. B-Raf is a serine/threonine kinase, which phosphorylates the mixed kinase MEK (MAP/ERK kinase, phosphorylates tyrosine and threonine residues) in the Ras/Raf/MAPK signaling pathway, which in turn leads to activation of the MAP kinase and activation of cytosolic and nuclear proteins that drive cell growth and differentiation [34]. In contrast to the oncogene KRAS, BRAF is not mutated in HCT-116 cells. As already illustrated for the oncogene KRAS, a highly significant increase in the secretion of EVs in the P14 fraction was also observed after the downregulation of the proto-oncogene BRAF (Figure 2a).

**Figure 6:**
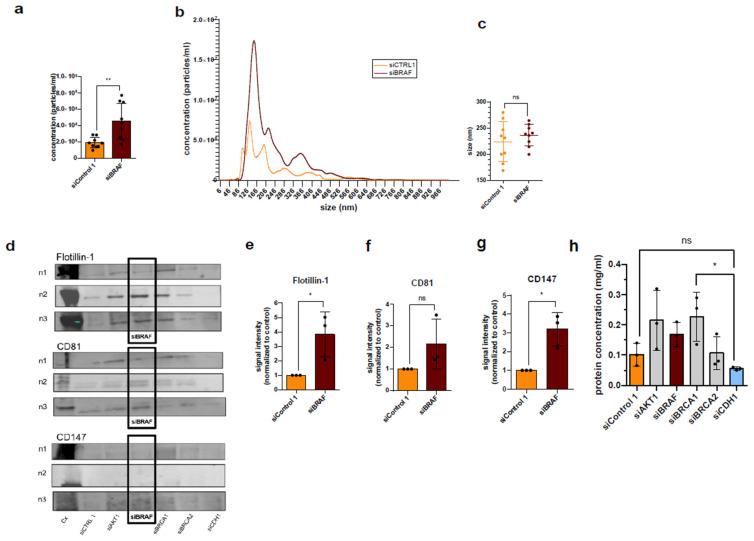
Knockdown of BRAF increases P14 EV secretion from HCT-116 cells. (**a**) Particle concentration measured by NTA shown as *t*-test, (**b**) size distribution of P14 EV siBRAF and siCtrl measured by NTA and (**c**) averaged sizes of P14 EVs after downregulation of BRAF compared to siCTRL, (**d**–**g**) marker distribution of Flotillin-1, CD81 and CD147 by Western blot and quantification of Flotillin-1 (47 kDa), CD81 (25 kDa) and CD147 (42–58 kDa), and (**h**) total protein concentration measured by BCA protein assay (enhanced protocol). Significance level: * *p* < 0.05, ** *p* < 0.01. Original images are provided in File S1.

A significantly increased secretion of EVs in the P14 fraction after the downregulation of BRAF in HCT-116 cells was observed (Figure 6a,b), and the markers of EVs in the P14 fraction (Flotillin-1 and CD147) were also significantly increased after RNAi of BRAF (Figure 6d–g). After the downregulation of BRAF, there was no significant increase in the size of the EVs in the P14 fraction (Figure 6b,c). Lastly, an increased total protein concentration in the P14 fraction was observed compared to the control (Figure 6h).

### 3.4. Knockdown of CTNNB1 and PKM Leads to Increased Secretion of P14 EVs

**Figure 7:**
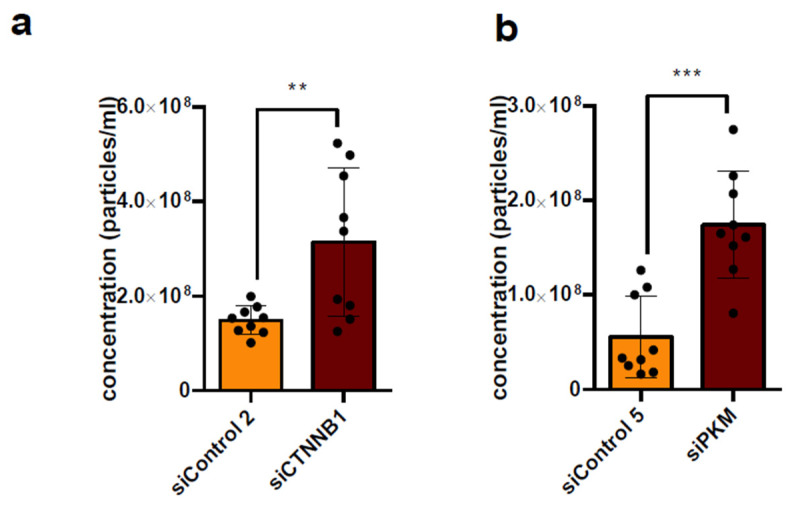
Knockdown of CTNNB1 and PKM increases P14 EV secretion from HCT-116 cells. (**a**) Particle concentration of P14 EVs from HCT-116 cells after downregulation of CTNNB1 and (**b**) PKM as measured by NTA. Analysis by unpaired *t*-test. Significance level: ** *p* < 0.01, *** *p* < 0.001.

In addition to the downregulation of the (proto-)oncogenes KRAS and BRAF, the downregulation of the (proto-)oncogenes CTNNB1 and PKM each also led to increased secretion of EVs in the P14 fraction (Figure 2e and Figure 7a,b).

CTNNB1, catenin (cadherin-associated protein) beta-1, is a proto-oncogene that encodes the protein β-catenin. β-catenin plays an important role in the regulation of cell adhesion and cell–cell contacts by connecting the cytosolic domain of cadherin-mediated adhesion complexes to the actin of the cytoskeleton. In addition, β-catenin is an important component of the Wnt signaling pathway, which promotes cell proliferation and is crucial for tissue homeostasis during embryonic development and in general. Upregulation of the Wnt signaling pathway leads to the accumulation of β-catenin cytosolically and especially nuclearly, where it supports the transcription of various oncogenes that drive carcinogenesis and tumor progression [35]. The HCT-116 cells used in this work carry an in-frame deletion; therefore, CTNNB1 functions as an oncogene in this case.

PKM, pyruvate kinase muscle, is a proto-oncogene that encodes the protein pyruvate kinase. Pyruvate kinase is an enzyme that catalyzes the crucial step of glycolysis by transferring a phosphate group from phosphoenolpyruvate (PEP) to adenosine diphosphate (ADP), thereby forming adenosine triphosphate (ATP). The ratio between the highly active tetramer form and the almost inactive dimer form of PKM determines whether carbons from glucose molecules are used for biosynthetic processes or for glycolytic ATP production. Due to alternative splicing, two isoforms of pyruvate kinase exist: M1 and M2 [36]. PKM is not mutated in HCT-116 cells.

### 3.5. Knockdown of CDH1 Leads to Reduced Secretion of P14 EVs

As the only candidate in this work, the downregulation of CDH1 led to a reproducible reduction in EVs in the P14 fraction, but without constant statistical significance in all aspects analyzed.

CDH1 is a tumor suppressor gene that encodes the transmembrane glycoprotein of the same name, cadherin-1, better known as “epithelial” cadherin (E-cadherin). This calcium-dependent cell adhesion protein forms homophilic cell–cell adhesions, i.e., an E-cadherin molecule binds an E-cadherin molecule from another cell. This can lead to contact inhibition of cells, modulated by growth inhibitory signal transduction pathways. Cadherins are connected to the actin filaments of the cytoskeleton via anchor proteins such as β-catenin. Cadherin-1 is found in all epithelia, regardless of whether they arise embryonically from ecto-, meso- or endoderm, since the CDH1 gene is already expressed in the blastomere stage. CDH1 therefore not only plays an important role in embryonic morphogenesis, but also in the stabilization of cell–cell contacts, in the maintenance of cell polarity, and as a signal transduction molecule. The complete absence or downregulation of CDH1 in the sense of a loss-of-function mutation is associated with uncontrolled cell growth and increased cell mobility [37]. In the HCT-116 cells used in this work, there is a mutation of CDH1 that leads to a truncated (shortened) protein.

**Figure 8:**
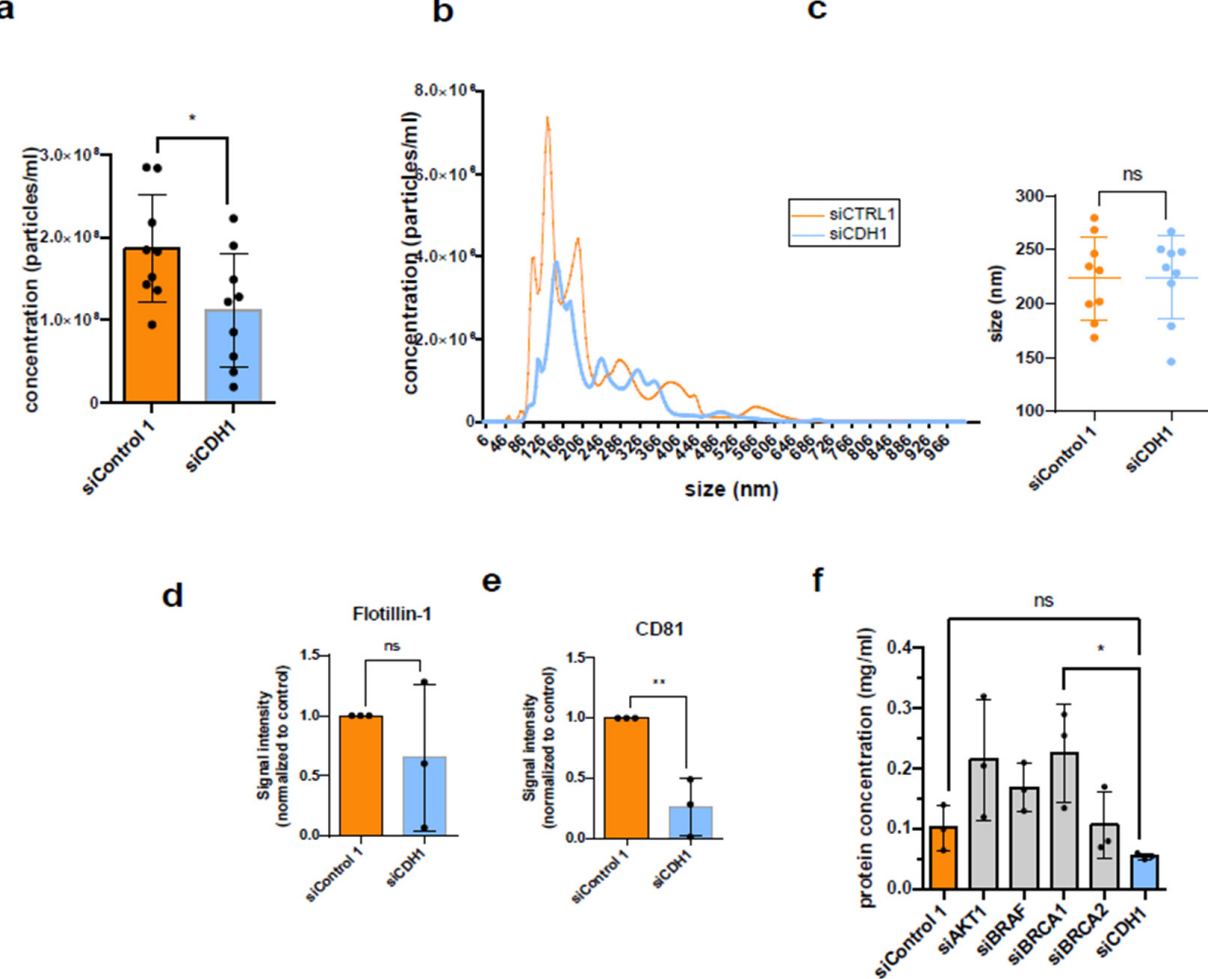
Downregulation of CDH1 decreases P14 EV secretion from HCT-116 cells. (**a**) Particle concentration of P14 EVs from HCT-116 cells after RNAi of CDH1 (with statistical significance determined by unpaired *t*-test); (**b**) size distribution and average sizes (**c**) of P14 EVs after knockdown of CDH1 compared to RNAi with non-targeting siRNA; (**d**,**e**) quantification of Flotillin-1 and CD81 from Western Blot from Figure 6d; (**f**) total protein concentration measured by BCA protein assay (enhanced protocol). Significance level: * *p* < 0.05, ** *p* < 0.01.

CDH1 proved to be particularly interesting in this work not only because of the reproducible reduction in EVs in the P14 fraction after CDH1 downregulation (Figure 8a), but also because it was the only tumor suppressor gene that suggested an influence on the secretion of EVs in all experiments (even if this was not consistently statistically significant) (Figure 8b–f).

### 3.6. Cell Viability

In order to assess whether the increased EVs secreted after the downregulation of specific cancer-related genes could be apoptotic bodies, an ATP-based cell viability assay, the CellTiter-Glo One Solution Assay, was carried out. Apoptotic bodies are EVs that are only secreted in cells that undergo apoptosis and are not secreted by viable cells. They vary greatly in size (approx. 100–5000 nm in diameter) and, above all, clarifying their function is also part of current research efforts in the field.

Compared to cells treated with non-targeting siRNA, none of the knockdowns resulted in a significant loss of cell viability as measured by ATP luminescence using the CellTiter-Glo One Solution Assay (Figure 5). Significant reductions in viability were observed only when compared to untreated HCT-116 cells following the downregulation of EGFR, KRAS, PIK3CA, SOX2, and TP63, reflecting a minor effect of RNA interference itself. Knockdown of KRAS, CTNNB1, BRAF, PKM, or CDH1 did not significantly affect cell viability compared to either control or untreated cells, and apoptosis was not directly assessed in this analysis.

## 4. Discussion

Extracellular vesicles (EVs) play a cardinal role in cell-to-cell communication in both physiological and pathological processes. In cancer, EVs have been shown to support tumor initiation as well as tumor growth, interaction with the tumor microenvironment, metastasis, and even drug resistance. Their potential as biomarkers as well as therapeutical targets is the content of current research efforts within the field [38].

The detailed molecular mechanisms of EV secretion in cancer remain unknown, whereas the fact that cancer cells secrete significantly more EVs compared to physiological cells is widely accepted. Metabolic processes have been suspected to play a role in tumor EV secretion, and singular tumor mutations representing either oncogenic or tumor suppressor functions have been linked to EV secretion in cancer [39]. But thus far, no systematic approach looking at the role of (proto-)oncogenes and tumor suppressor genes regarding EV secretion has been undertaken. This is why, in this study, a screening of 30 very common tumor mutations was conducted regarding their loss-of-function-mediated impact on EV secretion in the colorectal carcinoma cell line HCT-116.

Overall, downregulation of those 30 cancer-related genes with siRNA lead mainly to an increase in EV secretion, with a few exceptions (decreased secretion of P14 EVs after knockdown of *CDH1*, *GSTP1*, *MDM2*, *MET*, *MYC*, and *TGFB1* and decreased secretion of P100 EVs after RNAi of *AKT1*, *CDKN2A*, *CTNNB1*, and *KRAS*). A generic effect of neither (proto-)oncogenes nor tumor suppressor genes on the secretion of HCT-116 cells could not be found, whereas singular genes and associated signaling pathways appeared particularly interesting.

Downregulation of both the oncogene KRAS and the protooncogene BRAF led to a significant increase in P14 EV secretion, increased levels in known EV markers, and increased total protein levels in the P14 fraction, suggesting a potential impact of these (proto-)oncogenes on EV secretion. Driving around one third of all human cancers, mutated Ras proteins (isoforms n-Ras, h-Ras, k-Ras) controlling crucial cell proliferation and survival pathways like the Ras/Raf/MAPK pathway lead to uncontrolled tumor growth. A recent study implicates a KRAS-dependent secretion of small EVs driven by Rab13 [40]. In this particular study, knockdown of mutant KRAS led to a significant decrease in sEVs, concordant to the decrease in P100 EVs in our study (even though it was not statistically significant).

Small EVs secreted by mutant KRAS-expressing colon cells have also been shown to enhance the invasiveness of recipient cells relative to sEVs isolated from wild-type KRAS-expressing cells. Demory Beckler et al. demonstrated the presence of mutant KRAS on sEVs, as well as the ability of sEVs transferring mutant KRAS to only wild-type KRAS-expressing cells and thereby enhancing originally wild-type KRAS cell growth [32]. Their demonstrated KRAS-dependent shift in the exosomal proteome appears intriguing considering our findings of a significantly higher protein content in the P14 fraction as well as a significant shift towards, on average, bigger P14 EVs after the downregulation of KRAS.

An average shift in particle size towards bigger EVs also poses the possibility of having isolated apoptotic bodies instead of large EVs (i.e., microvesicles). Knockdown of a driver mutation leading to apoptosis would surely make sense, but the ATP-based cell viability assay revealed no significant decrease in cell viability compared to RNAi with non-targeting siRNA under the same knockdown conditions used during EV isolation. Increased P14 EV release after *KRAS* or *BRAF* knockdown may reflect the activation of intracellular stress responses caused by disrupted oncogenic signaling. Such stress has been linked to enhanced EV release and altered EV composition, potentially influencing tumor–microenvironment communication [41].

Wnt-dependent secretion of EVs also appears possible. Both wild-type β-catenin [26,42] and oncogenically mutated β-catenin [43] have already been detected on EVs. The E-cadherin-dependent, sEV-mediated reduction in intracellular β-catenin has been described as a mechanism to antagonize the Wnt signaling pathway. In HCT-116 cells, both the oncogene β-catenin (CTNNB1) and the tumor suppressor gene E-cadherin (CDH1) are mutated. In this study, downregulation of the oncogene CTNNB1 resulted in a significantly increased secretion of EVs in the P14 fraction, whereas downregulation of the tumor suppressor gene CDH1 led to a (not consistently significant) reduction in EVs in the P14 fraction. In the P100 fraction, however, EVs were (not significantly) decreased after CTNNB1 downregulation and (not significantly) increased after CDH1 downregulation, each compared to RNAi with non-targeting siRNA.

Auber and Svenningsen identified a correlation between the secretion rates of EVs in various blood cells and their mitochondrial metabolism. In their study, reactive oxygen species (ROS) were identified as responsible for the secretion rate of EVs, pointing out that tumor cells, which generally secrete more EVs than non-tumor cells, possess particularly high cellular levels of ROS. They furthermore demonstrated the correlation between this and the activity of mitochondrial complex I, but only in physiological, non-degenerated human blood cells from healthy individuals [44]. However, mitochondrial metabolism was discussed as a possible mechanism for controlling the secretion rate of EVs in tumor cells and should therefore be mentioned here.

Wei et al. were also able to establish a connection between the metabolism of tumor cells and their secretion of EVs. They illustrated a correlation between aerobic glycolysis and sEVs secretion. Furthermore, their study demonstrated the dependence of sEVs secretion on the dimerization of the enzyme PKM2. PKM2 downregulation resulted in a significant reduction in the secretion of EVs in their study [45]. PKM2 is one of the two isoforms of the protein encoded by PKM. Contradictorily, the downregulation of the PKM gene in our study did not lead to a reduction in concentration in either the P14 or P100 fraction.

Despite the novel insights provided by our screening approach, several limitations should be acknowledged. First, the findings are based only on the HCT-116 colorectal cancer cell line, which has a specific mutational background and may not represent other tumor types or even other colorectal cancer models. Even though we used siTOOLs RNAi, which applies pools of 30 distinct siRNAs per target to minimize off-target effects and enhance knockdown efficiency through cooperative silencing, residual off-target effects or incomplete knockdown cannot be fully excluded, which could influence EV secretion independent of the intended target. While transient knockdown lacks the sustained effects of stable silencing, it avoids cellular adaptation and better reflects immediate gene-specific effects on EV secretion. However, the study does not yet address the biological relevance of reduced EV output. Future work should test whether targeting these pathways can disrupt tumor–microenvironment interactions and related tumor-promoting processes. While nanoparticle tracking analysis and protein quantification were used to assess EV levels, the results may be affected by variations in cell number, viability, or metabolic state. Importantly, only loss-of-function effects were investigated, whereas gain-of-function mutations—common in cancer—may influence EV secretion differently. Moreover, the study assessed EVs only at a single time point (at 72 h post-siRNA transfection). As with many screening approaches, an initial fixed time point was chosen to identify candidate genes, which can then be followed by time-course analyses for selected hits to capture dynamic or delayed effects. Similarly, we did not include functional assays to determine the biological relevance of the observed changes in EV quantity or composition. Finally, the monolayer culture system used here lacks components of the tumor microenvironment that are known to modulate EV biology, such as stromal or immune cells. These limitations underscore the need for further validation across multiple cell lines and functional assays and in more physiologically relevant models.

## 5. Conclusions

Nonetheless, the study provides important initial insights into the regulation of EV secretion by tumor-related genes. The systematic screening revealed that neither (proto-)oncogenes nor tumor suppressor genes exert a uniform effect on EV secretion in HCT-116 cells. However, specific candidates and signaling pathways—particularly KRAS and BRAF in the Ras/Raf/MAPK pathway and CDH1 and CTNNB1 in the Wnt pathway, emerged as promising targets for future investigation. Additionally, the potential role of tumor cell metabolism in modulating EV release offers a compelling direction for follow-up studies. Together, these findings lay the groundwork for a more detailed understanding of the molecular mechanisms controlling EV secretion in cancer.

## Figures and Tables

**Table 1:** Candidates and their mutational status in HCT-116 cells.

Candidate ^1^	Gene Name	Mutational Status in HCT-116-Cells ^2^
*AKT1*	*v-akt murine thymoma viral oncogene homolog 1*	None
*BRAF*	*v-raf murine sarcoma viral oncogene homolog B1*	None
*BRCA1*	*breast cancer susceptibility gene 1*	None
*BRCA2*	*breast cancer susceptibility gene 2*	None
*CDH1*	*cadherin 1*, *type 1*, *E-cadherin (epithelial)*	X441_splice, H121Tfs*94 truncating mutation (putative driver mutation)
*CDKN2A*	*cyclin-dependent kinase inhibitor 2A (p16(INK4a)) gene*	None
*CTNNB1*	*catenin (cadherin-associated protein)*, *beta 1*	S45 del Inframe mutation (putative driver mutation)
*EGFR*	*epidermal growth factor receptor (erythroblastic leukemia viral oncogene homolog 1)*	None
*ERBB2*	*erythroblastic leukemia viral oncogene homolog 2*	None
*ERBB3*	*erb-b2 receptor tyrosine kinase 3*	Q261* truncating mutation (putative passenger mutation)
*GSTP1*	*glutathione S-transferase pi 1*	Missense mutation (putative passenger mutation)
*HIF1A*	*hypoxia inducible factor 1 alpha subunit*	None
*JAK2*	*janus kinase 2*	None
*KRAS*	*kirsten rat sarcoma 2 viral oncogene homolog*	G13D missense mutation (putative driver mutation)
*MDM2*	*mdm2 p53 binding protein homolog*	None
*MET*	*met proto-oncogene* *(hepatocyte growth factor receptor)*	L238Yfs*25 truncating mutation (putative passenger mutation)
*MLH1*	*E.coli MutL homolog gene*	S252* truncating mutation (putative driver mutation)
*MTOR*	*mechanistic target of rapamycin*	None
*MYC*	*v-myc myelocytomatosis viral oncogene homolog (avian)*	Amplification
*NFKB1*	*nuclear factor kappa B subunit 1*	None
*PIK3CA*	*phosphatidylinositol-4,5-bisphosphate-3-kinase*, *catalytic subunit alpha*	None
*PKM*	*pyruvate kinase*, *muscle*	H1047R missense mutation (putative driver mutation)
*PTEN*	*phosphatase and tensin homolog gene*	None
*PTK2*	*protein tyrosine kinase 2*	None
*SOX2*	*SRY (sex determining region Y)-box 2*	Amplification
*STAT3*	*signal transducer and activator of transcription 3 (acute-phase-response factor)*	Missense mutation (putative passenger mutation)
*TP53*	*tumor protein p53*	None
*TP63*	*tumor protein p63*	None
*TGFB1*	*transforming growth factor beta 1*	Missense mutation (putative passenger mutation)
*VEGFA*	*vascular endothelial growth factor A*	Y103H missense mutation (putative passenger mutation) None

^1^ (Proto-)Oncogenes are depicted in dark red, tumor suppressor genes in blue. ^2^ According to [27].

**Table 2:** Camera settings.

Camera Settings	P14	P100
Camera Level	13	14
Screen Gain	1	3

**Table 3:** Analysis settings.

Analysis Settings	P14	P100
Screen Gain	10	10
Detection Threshold	6	3

## Data Availability

Further data can be taken from S.M.P.’s medical dissertation (doi: 10.53846/goediss-11114).

## Supplementary Materials

The following supporting information can be downloaded at: https://www.mdpi.com/article/10.3390/diseases14030089/s1, Figure S1: Western Blot of siTOOLs Knockdown of CTNNB1 and BRAF; File S1: Original-images.

## Abbreviations

The following abbreviations are used in this manuscript:

ADP	Adenosine diphosphate
AKT1	v-akt murine thymoma viral oncogene homolog 1 (proto-oncogene)
ATP	Adenosine triphosphate
BRAF	V-raf murine sarcoma viral oncogene hom-olog B1 (proto-oncogene)
CDH1	Cadherin-1, E-cadherin (tumor suppressor gene)
CDKN2A	cyclin-dependent kinase inhibitor 2A (p16(INK4a)) gene (proto-oncogene)
CTNNB1	β-Catenin (oncogene)
DMEM	*Dulbecco’s Modified Eagle Medium*
EGFR	*Epidermal growth factor receptor* (proto-oncogene)
EV	Extracellular Vesicle
GAPs	GTPase-activating proteins
GDP	Guanosine diphosphate
GEPs	Guanine nucleotide exchange factors
GSTP1	Glutathione S-transferase pi 1 (tumor suppressor gene)
GTP	Guanosine triphosphate
KRAS	v-Kirsten rat sarcoma gene (oncogene)
MDM2	Mdm2 p53 binding protein homolog (proto-oncogene)
MET	Hepatocyte growth receptor gene (oncogene)
MYC	Myelocytomatosis viral oncogene (oncogene)
NTA	Nanoparticle tracking analysis
PEP	Phosphoenolpyruvate
PIK3CA	Phosphatidylinositol(-4,5-bisphosphate)3-kinase, catalytic subunit alpha
PI3K	phosphatidylinositol 3-kinase
PIP3	Phosphatidylinositol 3,4,5-triphosphate
PKM	*Pyruvate kinase*, *muscle* (proto-oncogene)
PtdIns	Phosphatidylinositols
RNAi	RNA interference
ROS	Reactive oxygen species
Small EVs	sEVs
SOX2	SRY (sex determining region Y)-box 2 (oncogene)
siRNA	Small interfering RNA
TGFB1	Transforming growth factor beta 1 (oncogene)
TP63	Tumor protein 63 (tumor suppressor gene)
