# Supplementary material for: Functional siRNA Screen Links Ras/MAPK and Wnt Pathway to EV Secretion in HCT-116 Colorectal Cancer Cells
Source: Diseases. 2026 Mar 2;14(3):89. doi: 10.3390/diseases14030089 (PMC13025452; doi:10.3390/diseases14030089)
Supplement: Supplementary file 1 [file diseases-14-00089-s001.zip › File S1 - Western Blots.pdf]

1.1. Western Blots from Fig 4 (P14 after KRAS Knockdown)

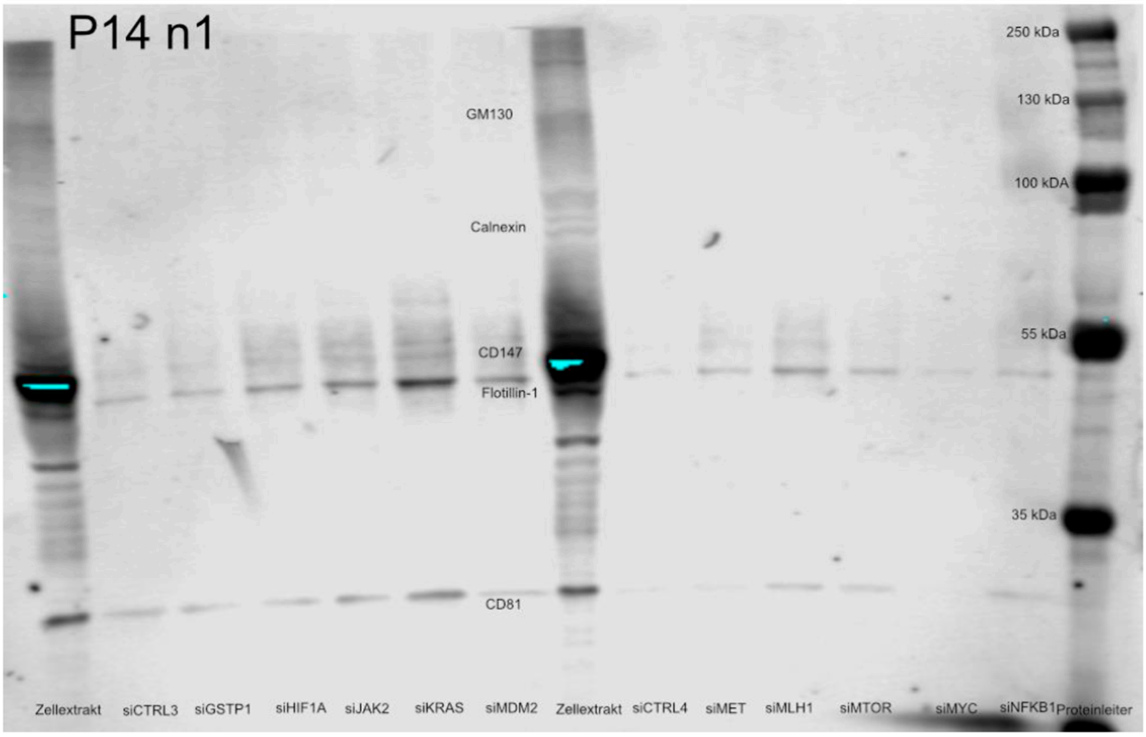

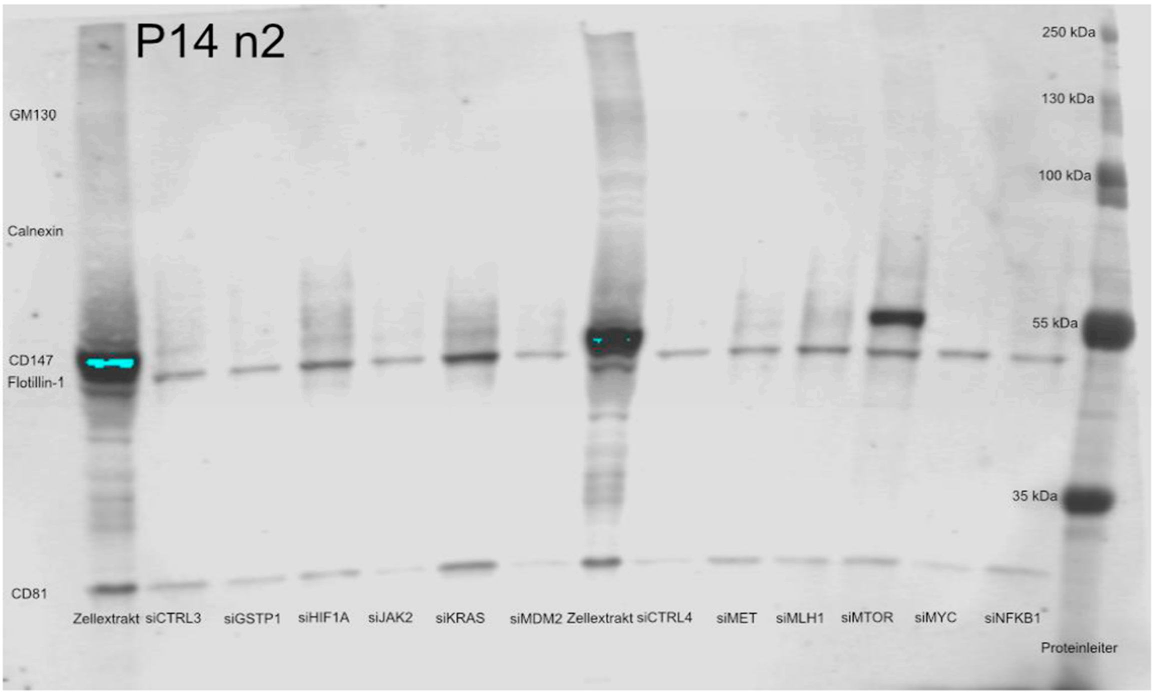

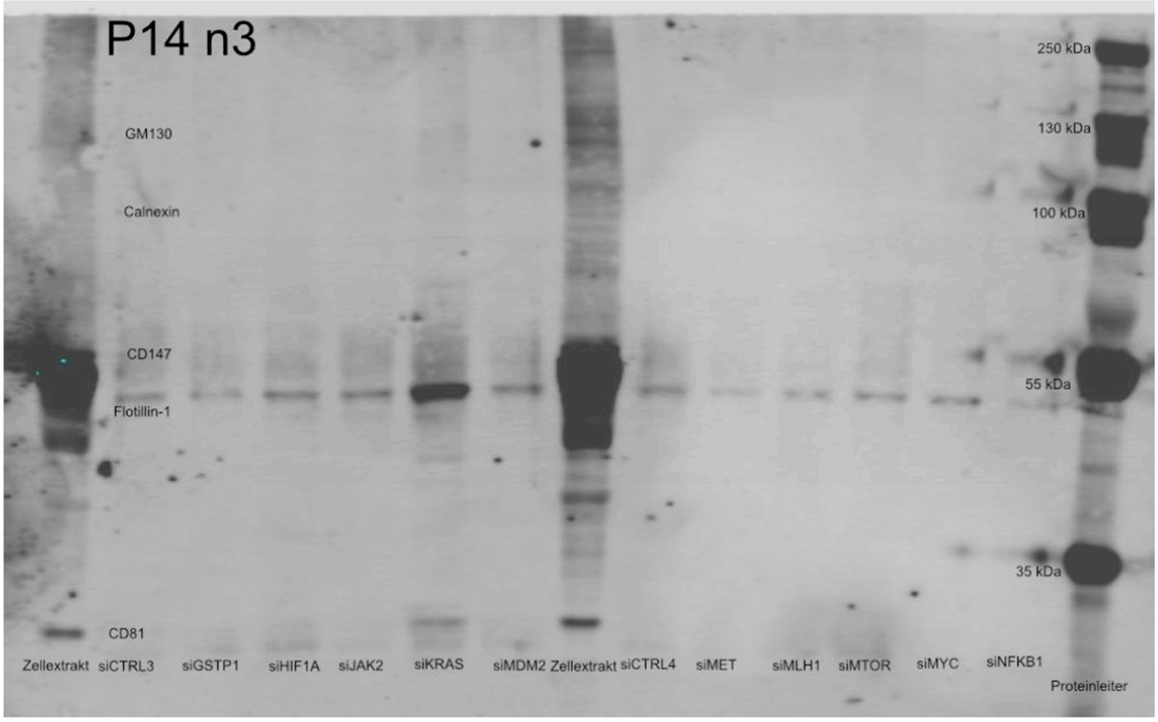
P14 n3
GM130
Calnexin
CD147
Flotillin-1
CD81
250 kDa
130 kDa
100 kDa
55 kDa
35 kDa
Zellextrakt
siCTRL3
siGSTP1
siHIF1A
siJAK2
siKRAS
siMDM2
Zellextrakt
siCTRL4
siMET
siMLH1
siMTOR
siMYC
siNFKB1
Proteinleiter

1.2. Western Blots from Fig 6 (P14 after BRAF-Knockdown) and 7 (P14 after CDH1-Knockdown)

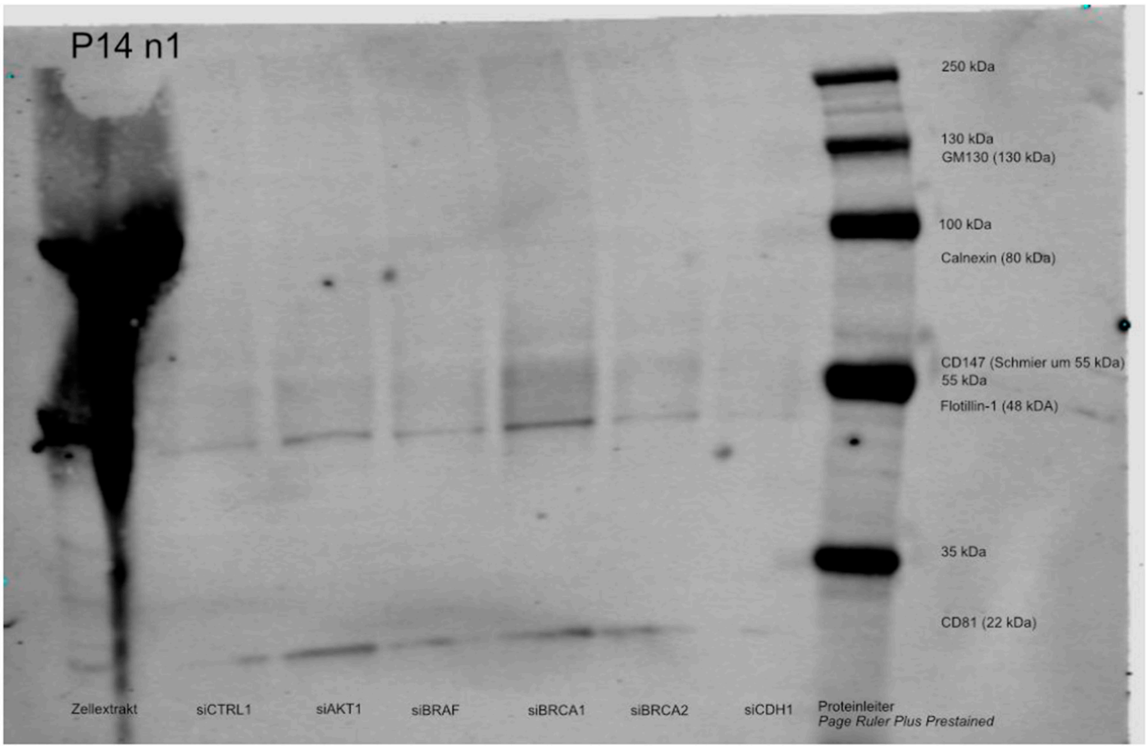

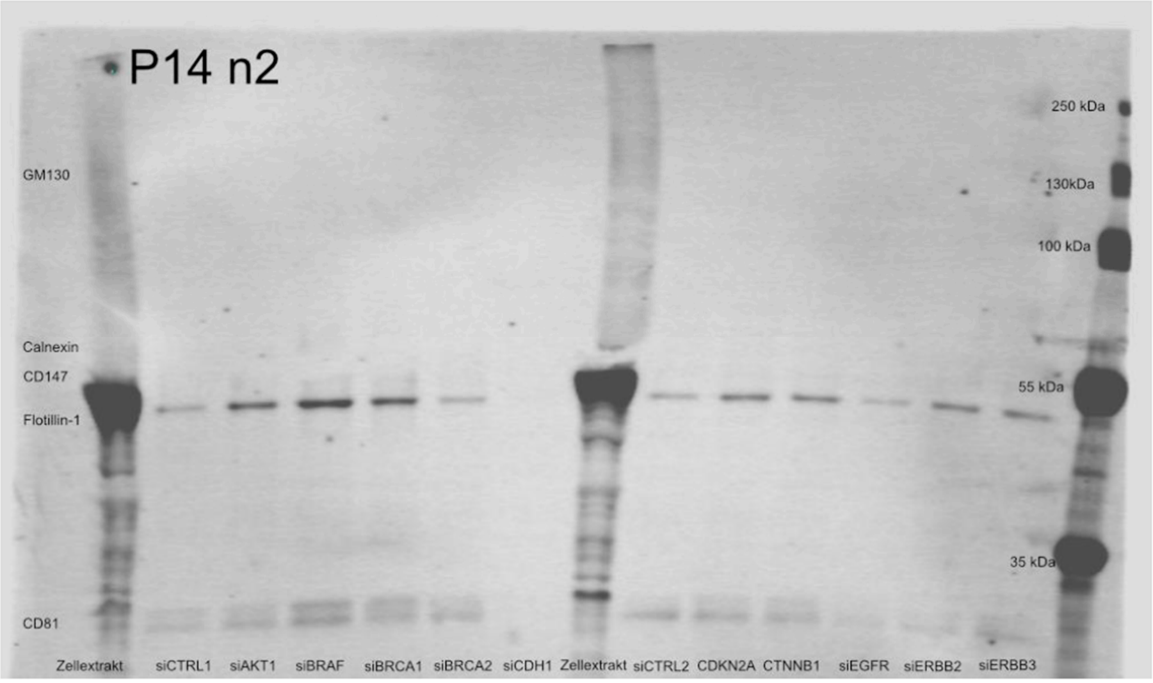

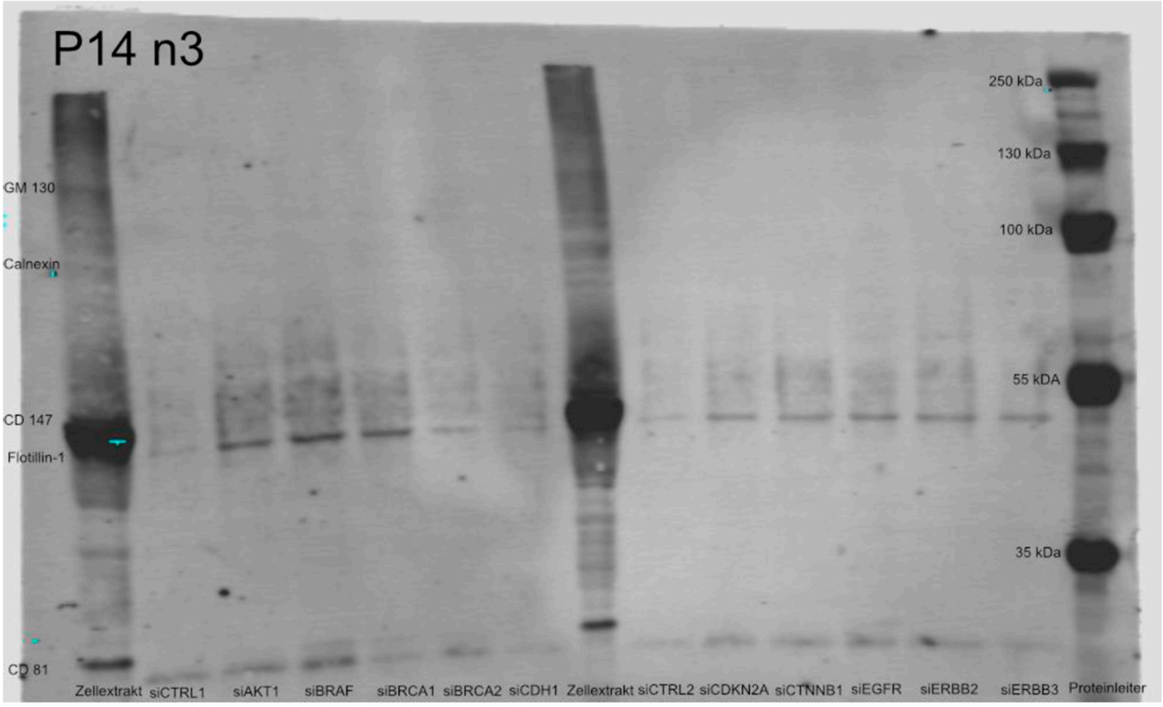
P14 n3
GM 130
Calnexin
CD 147
Flotillin-1
CD 81
250 kDa
130 kDa
100 kDa
55 kDA
35 kDa
Zellextrakt
siCTRL1
siAKT1
siBRAF
siBRCA1
siBRCA2
siCDH1
Zellextrakt
siCTRL2
siCDKN2A
siCTNNB1
siEGFR
siERBB2
siERBB3
Proteinleiter
