# Supplementary figures and images for: Functional siRNA Screen Links Ras/MAPK and Wnt Pathway to EV Secretion in HCT-116 Colorectal Cancer Cells
Source: Diseases. 2026 Mar 2;14(3):89. doi: 10.3390/diseases14030089 (PMC13025452; doi:10.3390/diseases14030089)

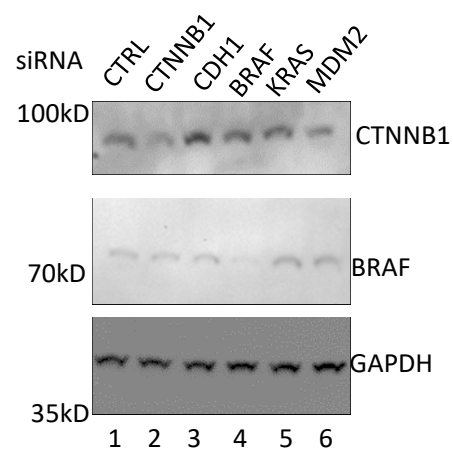

**Supplementary Figure S1 .Western Blot of siTOOLS Knockdown of CTNNB1 and BRAF**

Supplement: Supplementary file 1 [file diseases-14-00089-s001.zip › Figure S1.pdf]
